# Attachment strength of the subtidal seaweed *Sargassum horneri* (Turner) C. Agardh varies among development stages and depths

**DOI:** 10.1007/s10811-016-0869-5

**Published:** 2016-05-12

**Authors:** Min Xu, Shingo Sakamoto, Teruhisa Komatsu

**Affiliations:** Atmosphere and Ocean Research Institute, The University of Tokyo, 5-1-5, Kashiwanoha, Kashiwa City, Chiba 277-0882 Japan

**Keywords:** *Sargassum horneri*, Phaeophyceae, Attachment strength, Maturity stages, Depth

## Abstract

*Sargassum horneri* is one of the most important contributors to the rafts of floating seaweed in the waters off the coasts of northeastern Asia. These rafts serve as spawning and nursery grounds for many marine organisms, including Japanese saury and yellowtail. Thus, the details of the attachment/detachment mechanisms of *S. horneri* are of commercial significance for the aquaculture industry. Here, we describe variations in the attachment strength of *S. horneri* as it relates to its developmental stage and depth along a bottom gradient. We measured the attachment strength/dislodgement force of *S. horneri* samples with holdfast detachment in Shidagaura Cove, Shimoda, Japan, from December 2014 to May 2015. After we had determined the dislodgement forces required to detach thalli from the substratum (using a spring scale device) in the field, we transferred released individuals to the laboratory and measured selected morphological traits. Attachment strength was linearly related to the holdfast basal area when thalli were immature (prior to mid-March), but not when they were mature (mid-March to May). Thus, attachment strength was maintained through the reproductive phase and declined thereafter, allowing released thalli to join the drifting raft community. Rafting may be a mechanism by which the species expands its distribution range, as floating thalli continuing to shed germlings that are able to recruit when suitable habitat is encountered. Attachment strengths were greater in the shallows than in deeper water, reflecting the differences in wave forces experienced at different depths.

## Introduction

*Sargassum horneri* (Turner) C. Agardh is an annual brown seaweed species that is common along northwestern Pacific coastlines. Germlings released from the receptacles settle on different substrata in the spring–summer period and develop into visible immature thalli in autumn to winter as rhizoids grow forming holdfast and the lengths and weights of the developing seaweed increase. Thalli mature in spring. Dense forests develop on hard substrata, such as bedrock or boulders, surviving from depths of 1–3 m (Umezaki 1984; Komatsu et al. 2014). Populations grow down from the surface layer into the subtidal zone, reaching depths >7 m. On the Sanriku coasts of Japan, *S. horneri* forms stands of thalli that are attached to the bottom to a depth of 7 m and float upward, forming canopies on the sea surface (Komatsu et al. 2015). When mature thalli reach maximum lengths in the spring–early summer period, they are readily detached from the bottom by wave action with or without their holdfasts. When the holdfasts are torn off the substratum, the drag force created by waves has exceeded the attachment strength of the holdfast (Yoshida 1963). The thalli have many gas-filled vesicles that provide buoyancy for the floating canopy. Detached thalli float on the surface and often form rafts of tangled seaweed (Komatsu et al. 2006). A large number of floating *S. horneri* rafts occurred in the southern Yellow Sea drifting from south to north (Cai et al. 2014), and they were shown by genetic markers to be from Zhejiang province, China and Jiangling, Korea (Chen et al. 2016).

Floating seaweed rafts provide important ocean habitats for a diversity of fish (Senta 1986; Cho et al. 2001; Thiel and Gutow 2005). In the spring–early summer period, most of the floating rafts on Japanese coastlines contain detached thalli of *S. horneri* (Yoshida 1963; Ikehara 2004), and in the East China Sea, the floating rafts contain only *S. horneri* fronds (Komatsu et al. 2007, 2009, 2014; Mizuno et al. 2014), on which commercially important pelagic fish lay their eggs. These fish species include flying fish, Pacific saury (C*ololabis saira*), and Japanese halfbeak (*Hyporhamphus sajori*), which spawn egg-bearing filaments that attach to the floating seaweed rafts (Komatsu et al. 2009). Juveniles of yellowtail (*Seriola quinqueradiata*) and Japanese horse mackerel (*Trachurus japonicus*) find habitat in the floating seaweed rafts (Senta 1986). The Japanese yellowtail aquaculture industry captures these wild juveniles along with the floating rafts they inhabit and uses them to stock rearing facilities. This procedure is necessary because carnivorous juveniles of yellowtail prevent marine farmers from rearing the species from eggs. The farmers call yellowtail juveniles *mojyako*, *mo* and *jyako* meaning seaweed and juveniles in the Japanese language, respectively. A supply of floating *S. horneri* rafts is crucial for this industry. Hence, information on the wave forces required to detach the thalli is relevant to this form of aquaculture.

Kawamata (2001) studied the dislodgement of *Saccharina japonica* (Areschoug) Lane, Mayes, Druehl & Saunders on wave-sheltered and wave-exposed coasts. He transplanted individuals from wave-sheltered areas to wave-exposed areas, where thalli were quickly detached. Thus, the wave force environment experienced by seaweeds early in their life most likely affects the strength of their attachment to hard substrata. Sugawara et al. (1998) found that the attachment strength of *Eisenia bicyclis* (Kjellman) Setchell in shallow water exceeded that of *Ecklonia cava* Kjellman attached in deeper water at the same study site. Sugawara et al. (1998) postulated that the disparity in attachment strengths reflected differences in wave force as a function of depths. Drag forces are greater in shallower water. In an expectation that attachment strength of *S. horneri* adapts to environment pressures, we examined the attachment strength of *S. horneri* along a depth gradient.

Environmental conditions, such as storminess, season, and water temperature, may affect the attachment strength of algae. Milligan and DeWreede (2000) found a statistically significant difference in the attachment strengths of individuals of the intertidal seaweed species *Hedophyllum sessile* (C. Agardh) Setchell in a pre-storm group and those in a post-storm group. As storminess changed by season, they concluded that there was a seasonal effect on attachment strength that increased resistance to storms. Thus, we postulated that the attachment strength of *S. horneri* might change among growth stages and months.

To improve our understanding of factors influencing the supply of floating *S. horneri* rafts in the zones extending seaward from the coast to offshore waters, we studied attachment strengths of thalli as a function of growth stage, maturity stage, and depth. The analyses were performed in situ.

## Materials and methods

### Study site and sampling

The study site (34° 39′ 58″ N,138° 56′ 31″ E) was located in Shidagaura Cove, Shimoda, Izu Peninsular, Japan, which contains a wave-sheltered rocky shore close to the Shimoda Marine Research Center, Tsukuba University. Field surveys were conducted at intervals of *ca*. 1 month during the period of 15 December 2014 through 28 May 2015. During each survey, SCUBA divers randomly (random among thallus length and depth) sampled *ca*. 20–40 individuals of *S. horneri* that were attached at depths between 0 and 4 m below the mean low tide level (MTL) in Shimoda Port.

The force required to dislodge each individual was determined with spring scales measuring to 5 or 10 kg. A piece of twine was attached around each stipe immediately above the holdfast and looped onto the scale spring. The twine was pulled away from the rock surface until the holdfast released or broke, as described by Sugawara et al. (1998). The scale pointer recorded the maximum force exerted, and this measure was used as an indicator of the force required to: (i) dislodge the holdfast or the substratum surface to which it was attached or (ii) break the stipe or holdfast. This force was used as an indicator of attachment strength. The water depth in which the holdfast of each measured individual occurred was recorded during the survey. Through morphological examination, we classified dislodgement types into the following categories: (1) main stipe with an entire holdfast and (2) stipe detached from the substratum without a holdfast (Table 1). That main stipe detached from the substrate without a holdfast was precluded from further statistical analysis.

**Table 1:** Dislodgement force category assignments for *Sargassum horneri* thalli from December 2014 to May 2015

Month	Stem breakage	Dislodgement with holdfast^a^	Other	Total
December	0	29	1 (data lost)	30
January	1	28	1 (aggregated heptera)	30
February	4	25	1 (substrate: crustacea and coralline algae)	30
March	2	17	1 (attached to *E. bicyclis* holdfast)	20
April	2	17	1 (data lost)	20
May	1	38	1 (data lost)	40

^a^ Thallus dislodgement force data were used in the statistical tests

Thalli collected by the divers were individually numbered and transported to a laboratory where we obtained measurements of linear size and the weights of the thallus. When a whole holdfast or part of a holdfast remained on the substratum after testing attachment strength, we removed it from the substratum for measurements of area and weight. Samples were kept at −30 °C in a freezer until they were analyzed. After thawing, we measured thallus length from the bottom of the holdfast to the top of the longest lateral branch (with an accuracy of ±1 cm) using a 30-cm ruler. We used a balance to measure the wet weights of the thalli (with an accuracy of ±2 g) after absorbing surface water with a paper towel. The basal outline of each holdfast was drawn onto tracing paper with a pencil and digitized using an image scanner (GT-X970, Epson). The area of each holdfast was calculated using image analysis software, ImageJ 6.4 (NIH, USA, http://imagej.nih.gov/ij), following calibration with a square of known area (5 cm × 5 cm).

### Statistical analyses

We used one-way ANOVA to detect significant differences in measured attachment strength as a function of depth at 1-m depth intervals. Data were log transformed to fit the assumptions of normality and homoscedasticity required by this parametric procedure. Pearson correlation was applied in relationships among morphological traits and dislodgement force.

## Results

### Changes in dislodgement force by month

**Fig. 1:**
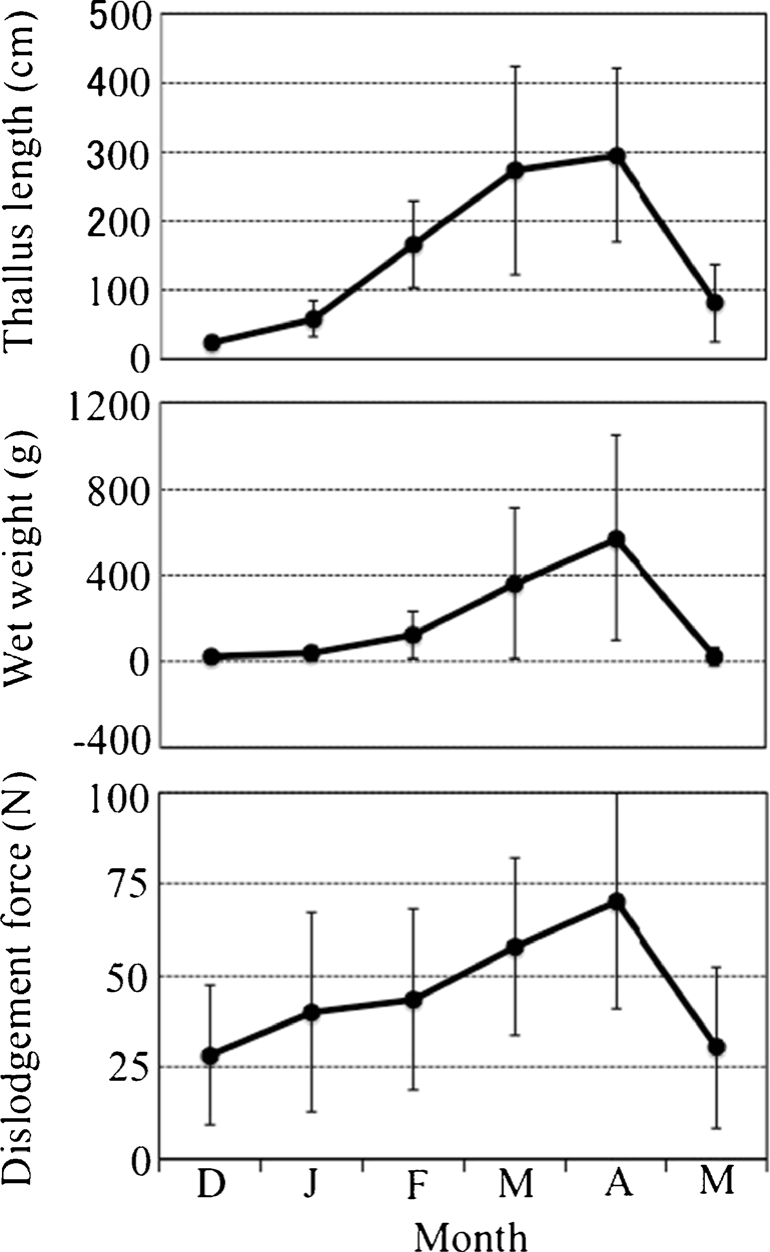
Mean thallus lengths (cm) and wet weights (g), and dislodgement forces (N) measured monthly from December 2014 through May 2015. Values are means ± SD

**Fig. 2:**
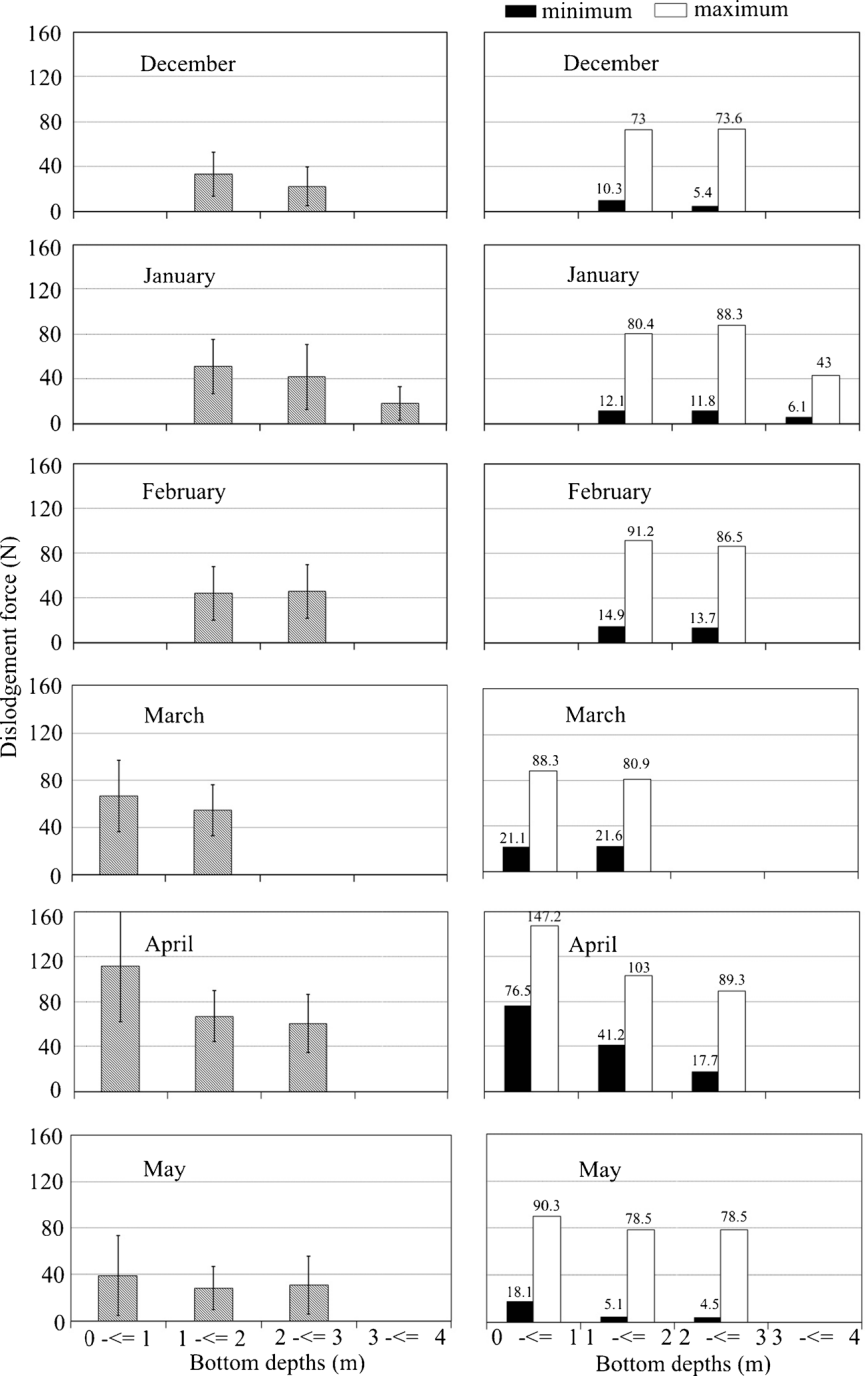
Mean (± SD) (*left column*), minimum (*filled bar*), and maximum (*open bar*) dislodgement forces (N) (*right column*) measured along the depth gradient from 0 to 4 m below the mean tide level for each month from December 2014 through May 2015

The mean force required for dislodgement of the samples increased from December 2014 to April 2015 as thalli increased in weight (Fig. 1). However, values decreased sharply in late May 2015. The mean wet weight of the samples was highest in April 2015 and declined rapidly in late May 2015 as most of the larger thalli disappeared. The maximum attachment strength in each month was stable (*ca*. 90 N). Two of the attachment strengths in April were >100 N, but values for most of the other samples in that month were ≤90 N and similar to those in other months. The minimum attachment strengths were in the range of 4.5 to 21.1 N (Fig. 2).

### Dislodgement force variation across a depth gradient

Dislodgement force did not vary significantly with depth (*p* > 0.05, Table 2) (the ratio of between-group variance to within-group variance was less than the statistical *F* variable *F*_crit_ (*p* = 0.05), indicating that there was no statistically significant difference among depth groups). However, mean dislodgement forces of the shallow samples tended to exceed those in deeper water from December to May (Fig. 2). Both maximum and minimum dislodgement forces seemed to increase from December to February across the depth gradient.

**Table 2:** One-way ANOVA testing for statistical differences in dislodgement force along the depth gradient from December 2014 to May 2015

	Analysis of variance (one-way)					
Stage	Month	Source of variation	MS	F	*p* value	F crit
Growth	15 December	Between groups	0.20	2.72	0.11	4.24
		Within groups	0.07			
	28 January	Between groups	0.31	2.70	0.09	3.44
		Within groups	0.11			
	25 February	Between groups	0.02	0.33	0.57	4.32
		Within groups	0.07			
Mature	31 March	Between groups	0.01	0.14	0.71	4.67
		Within groups	0.05			
	28 April^a^	Between groups	0.03	0.72	0.42	4.84
		Within groups	0.04			
	28 May	Between groups	0.36	0.51	0.60	3.29
		Within groups	0.71			

^a^Only measurements collected for depths of 1–2 and 2–3 m were included in the analyses of thalli sampled on 28 April 2014 because the sample size for the 0–1 m depth range on that date was too small for inferential testing (two samples only)

### Monthly changes in dislodgement force in relation to thallus growth

**Fig. 3:**
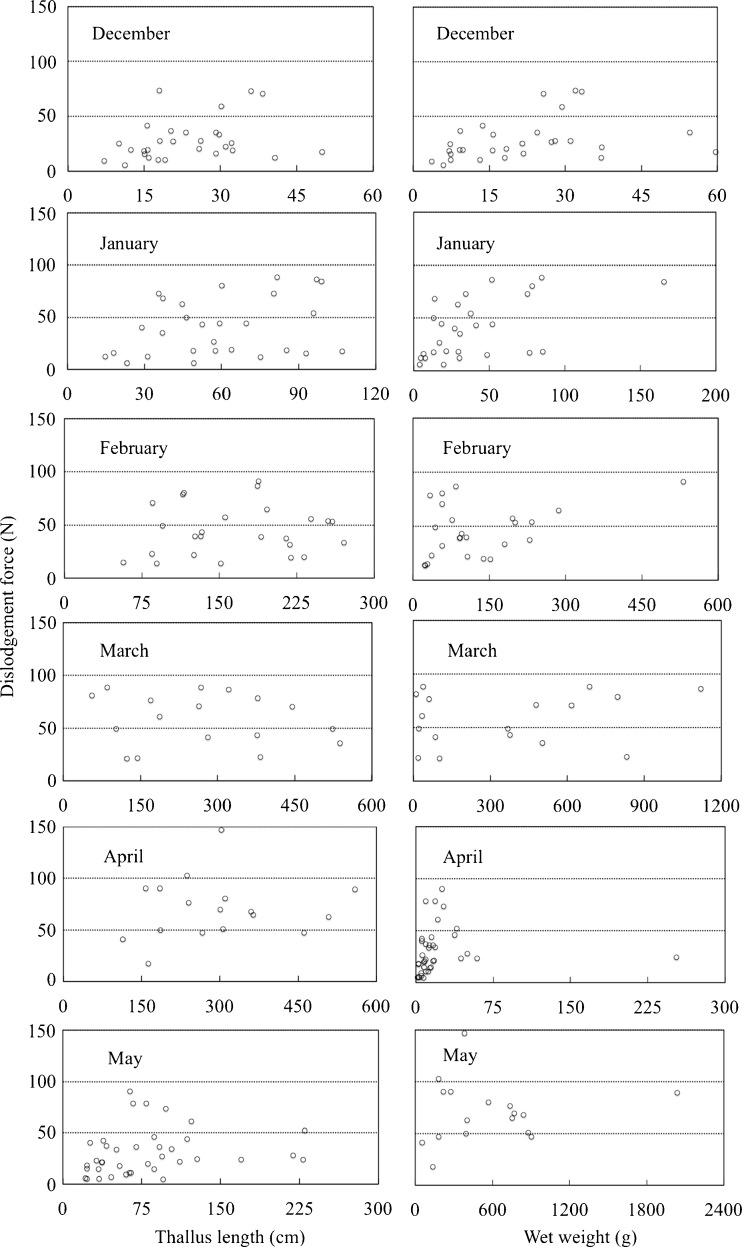
Relationships between dislodgement force (N) and (i) thallus length (cm) (*left column*) and (ii) wet weight (g) (*right column*) for each month from December 2014 through May 2015

**Fig. 4:**
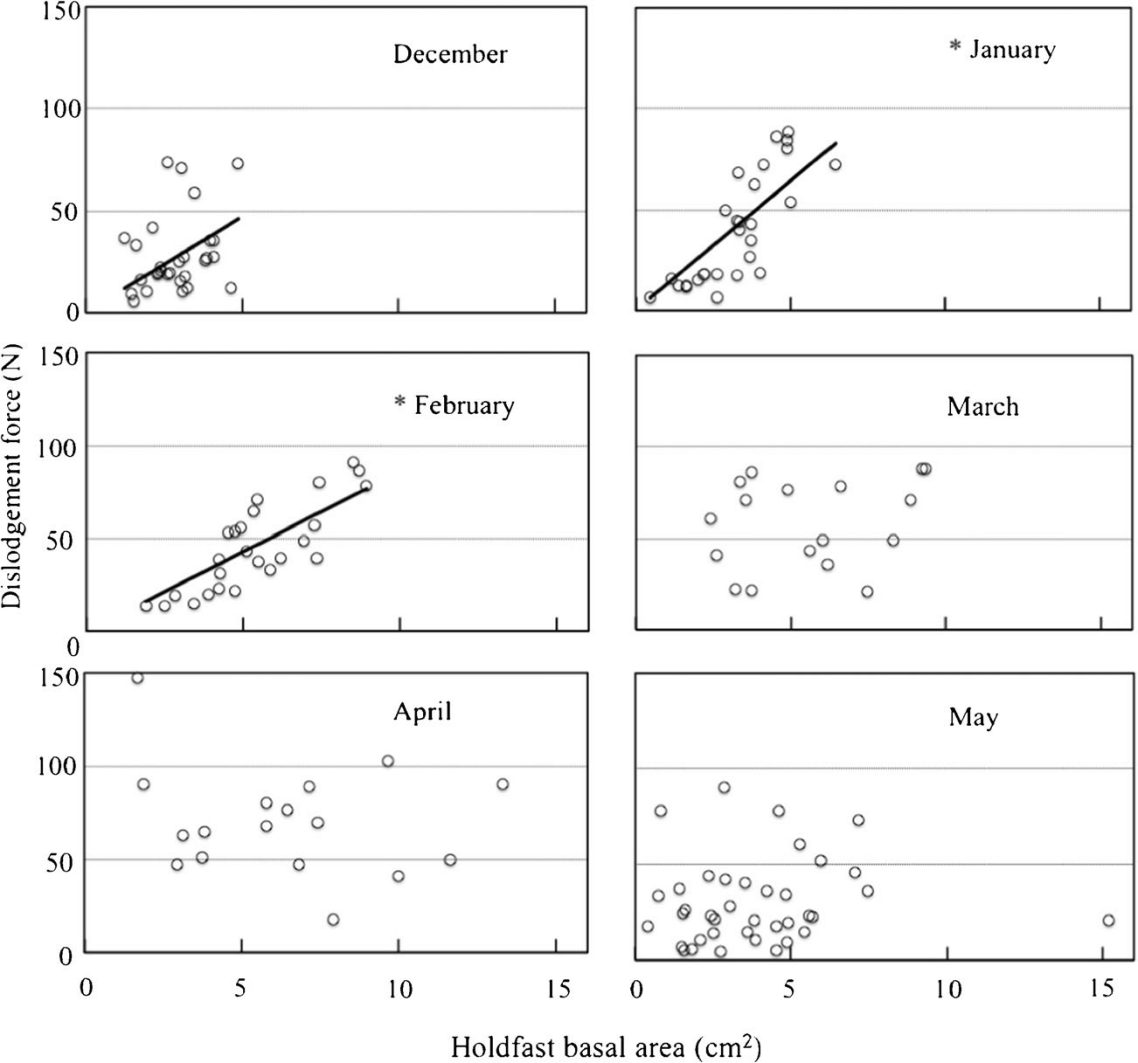
Relationship between dislodgement force (N) and holdfast basal area (cm^2^) for each month from December 2014 through May 2015. *Solid lines* are regression fits. *Asterisk* indicates significant linear correlations in January 2015 (*r* ^2^ = 0.86, *p* < 0.001) and February 2015 (*r* ^2^ = 0.81, *p* < 0.001)

Thallus length and wet weight had no relationship with dislodgement force across months (Fig. 3). However, holdfast basal area was linearly related to dislodgement force in January and February, though not from March to May (Fig. 4).

### Monthly changes in holdfast basal area across a depth gradient

**Fig. 5:**
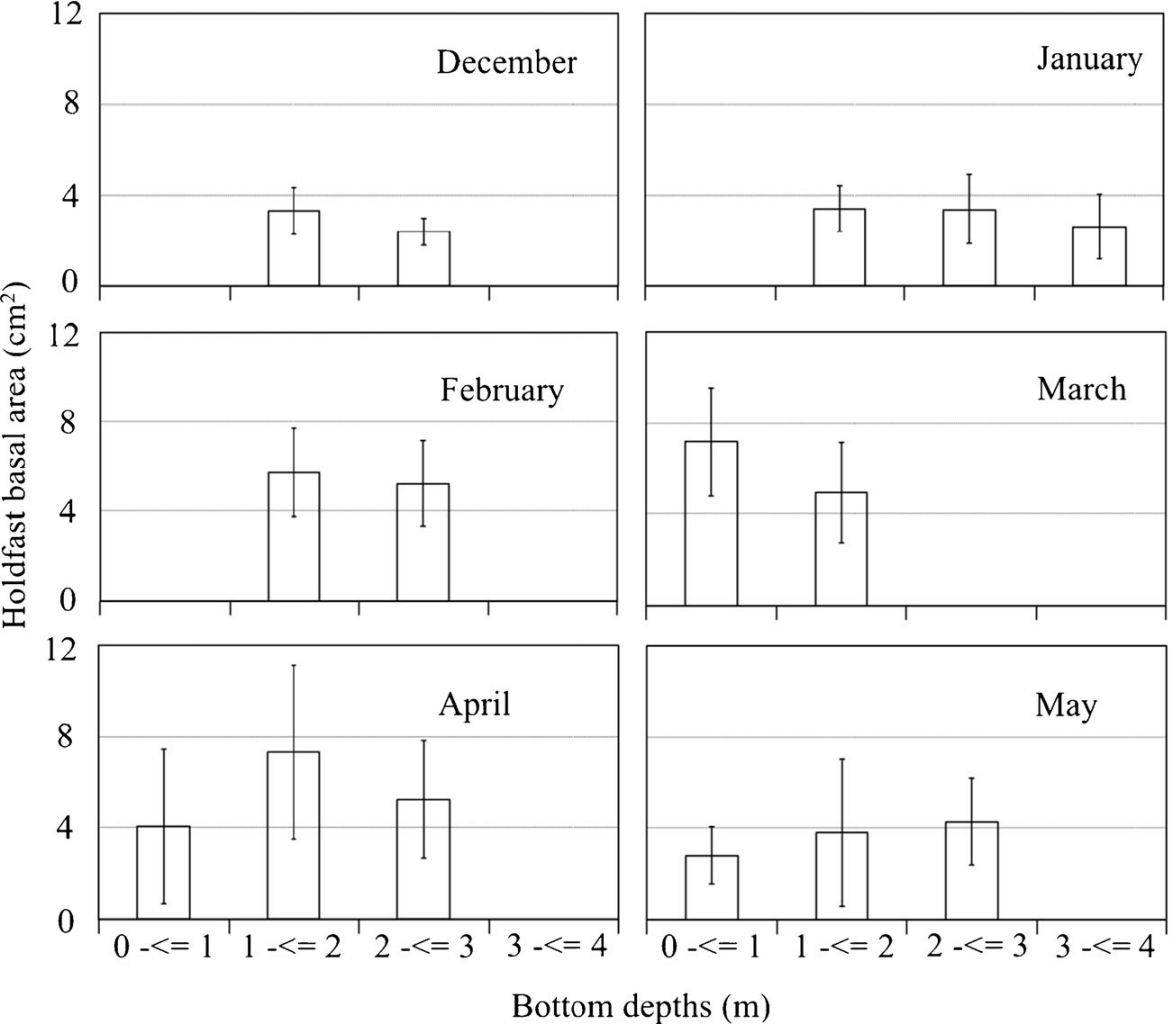
Mean holdfast basal areas (cm^2^) (open bar) (±SD) measured along the depth gradient from 0 to 4 m below the mean tide level for each month from December 2014 through May 2015

The mean monthly holdfast basal area of shallow water samples exceeded that of deeper samples from December to March, but not in April or May (Fig. 5).

## Discussion

Holdfast basal area was strongly related to dislodgement force from December to February. Thallus length and wet weight were not related to this force. The holdfast area in contact with the substratum fastens the thallus in position and accounts for the attachment strength of each individual. Kawamata (2001) found that the holdfast wet weight of *S. japonica* in a wave-exposed area was linearly related to attachment strength (*r* ^2^ = 0.57, *p* < 0.05). Holdfast performance is crucial to the ability of a seaweed to remain fastened in place on the bottom. Holdfast wet weight is likely correlated with holdfast basal area. We found that the attachment strength of *S. horneri* was proportional to holdfast area.

**Fig. 6:**
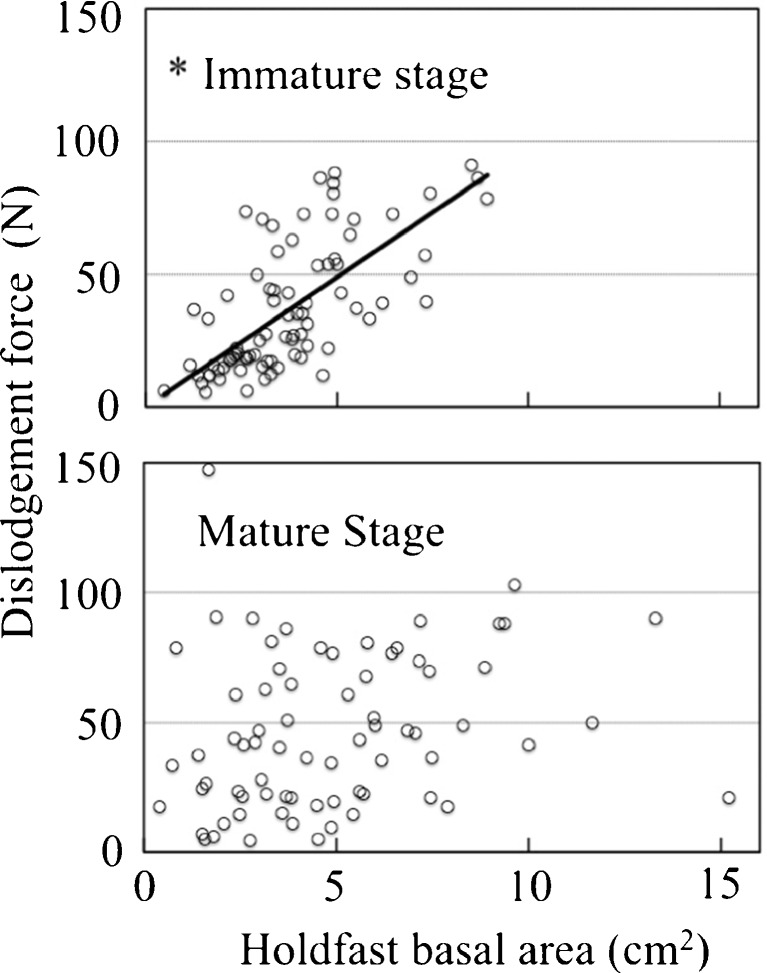
Relationship between dislodgement force (N) and holdfast basal area (cm^2^) for immature and mature thalli (December 2014–February 2015 and March 2015–May 2015, respectively). *Solid lines* are regression fits. *Asterisk* indicates significant linear correlation in the immature stage (*r* ^2^ = 0.67, *p* < 0.001)

Mikami (2007) set three permanent quadrats in an area of Shidagaura Cove where *S. horneri* was dominant and tagged all the stipes in each quadrat. She reported that mature stipes were found in mid-March but disappeared in May. Accordingly, samples in the December–February period were classified as immature and those occurring in the late March–May period were classified as mature. Holdfast basal area was proportional to attachment strength during the immature stage (*r* ^2^ = 0.53, *p* < 0.001) (Fig. 6), and we consider this relationship a morphological adaptation for thallus fixation.

*S. horneri* is an annual, dioecious species. Germlings are released over several days (3–4 days at a water temperature of 20 °C) after fertilization of eggs in the female receptacles (Koumoto and Tomiyama 1968). Koumoto and Tomiyama (1968) found that 25–50 % of eggs were released onto the surface of the receptacles during a first liberation period and that the remaining eggs were released 2 weeks later. Tahara (1913) reported that five egg release events occurred in *S. horneri* at intervals of 7–12 days during an approximately 1-month study on the Misaki Peninsular near Tokyo. Germlings were released from female receptacles on several occasions during the mature stage. The mean sedimentation velocity of *S. horneri* germlings was *ca*. 0.5 cm s^−1^ (germlings are heavier than seawater) (Okuda 1985). Since the receptacles were 3 m above the bottom, the germlings took *ca*. 10 min to reach the substratum with most settling around their parent thalli where the substratum and other environmental conditions are likely appropriate for growth and persistence. The fixation capability of the germlings is a significant contributory factor in population survival, but restricts the colonization of new habitats.

We found that holdfast basal area was not related to attachment strength in the mature stage from April to May. Floating adults and heavy germlings are common among *Sargassum* species. *Sargassum muticum* (Yendo) Fensholt was accidentally transported with seed oysters from Japan to France in the 1960s. Rueness (1989) reported that floating *S. muticum* rafts occurred along the Skagerrak coastline of Norway in 1984; individuals fixed to the bottom were not found until 1988. Detachment and floating of mature thalli may be regarded as a strategy for colonizing new territory, while germling fixation is a strategy for persistence in the natal habitat. Seaweeds with gas bladders commonly float out of the territories in which they recruited. Hernández et al. (2006) reported that sporophytes of drifting *Macrocystis pyrifera* (L) C. Agardh remained fertile with high germination success as long as the sori were present on the blades (sori persisted for 125 days). Rafting bull kelp (*Durvillaea antarctica* (Chamisso) Hariot) off north/central Chile was also found to be fertile (Tala et al. 2013).

In the early maturation stage, *S. horneri* stopped growing and invested energy into reproduction through the production of receptacles. Thalli remain fixed to the substratum. Hence, the first germlings were able to attach near their parents. A secure holdfast attachment is crucial at this phase. After release and fixation of early germlings, the attachment strength of mature *S. horneri* was not maintained, although sexual reproduction continued. Mature individuals started to lose their fixation capability and were readily dislodged when germling release had been completed. In late March, the individuals at our study site comprised a mixture of immature and mature thalli (Mikami 2007), which obscured any relationship between holdfast basal area and dislodgment force.

Most of the individuals had matured by April–May when holdfast basal area was not related to attachment strength in the Shidagaura Cove population. Attachment strength was markedly lower in May than it had been in April. The population in March contained individuals with no significant relationship between holdfast basal area and dislodgment force and others in which the two parameters were correlated; the two groups comprised mature and immature thalli, respectively.

Our dislodgment force measurements along the depth gradient also reflected maturity status. During the immature stage, attachment strength was highest in shallow water. Stewart (2006) transplanted *Turbinaria ornata* (Turner) J. Agardh between back reef and fore reef zones to investigate the adaptation of this species to wave-exposed environments. All of the samples transplanted from the back reef to the fore reef disappeared, but samples transplanted in the reverse direction were still alive when the experiment was terminated, suggesting that attachment strengths were highest in thalli that had developed in wave-exposed habitats. Kawamata (2001) obtained similar results when *S. japonica* thalli attached to plastic plates were transplanted between wave-protected and wave-exposed sites. The attachment strengths of *Stephanocystis hakodatensis* in wave-exposure sites exceeded those in wave-protected sites (Kuwahara et al. 1999). Thus, our findings were concordant with those of previous studies and indicate that *S. horneri* adapts to wave forces by increasing its holdfast attachment strength.

The force required to dislodge *S. hakodatensis* in wave-exposed sites ranged from 60 to 140 N (mean = 100 N) (Kuwahara et al. 1999). Milligan and DeWreede (2000) measured similar dislodgement forces (mean = ∼100 N) for the intertidal seaweed *H. sessile* (C. Agardh) Setchell. *S. horneri* generally grows in wave-protected waters, where the disparity of maximum and minimum dislodgement forces ranged from 67 to 85 N, varying by month. Our measurements were comparable to those of other studies that have examined seaweed species living in the intertidal zone or at wave-exposed sites. The lowest dislodgement forces that we determined for *S. horneri* thalli were in the range of 4.5 to 21.1 N, varying by month. The dislodgement forces required to release *S. japonica* at wave-protected sites were also <20 N (Kawamata 2001). The thalli of *S. horneri* are not especially robust considering the large dimensions that they attain during a single growing season. This may explain the restricted occurrence of the species to wave-protected waters (Marui et al. 1981; Umezaki 1984; Terawaki 1986; Sun et al. 2008).

In conclusion, this is a novel study of seasonal variation in *S. horneri* attachment strength. During the immature stages of growth, holdfast basal area was a major determinant of attachment strength, required to prevent dislodgement by wave action, but this was not the case when thalli were mature. Tenacious attachment during reproduction ensures that germlings settle close to parents where habitat conditions are likely favorable for the species. Weak attachment at later stages allows thalli to drift and potentially colonize new habitats by releasing germlings during the rafting phase. Thus, the species appears to have a strategy for persistence in its home environment and another for increasing its distribution range.

## References

[CR1] Cai YC, Sun B, Ma JH, He PM, Zhang Q (2014). Molecular identification of floating *Sargassum horneri* in the southern Yellow Sea. Mar Fish/Haiyang Yuye.

[CR2] Chen J, Wang YC, Yu QR, Bi YH, He PM, Liu ZY, Qin S (2016). Molecular phylogenetic analysis of floating *Sargassum horneri* associated with green tides in coastal area of Qingdao. J Biol.

[CR3] Cho SH, Myoung JG, Kim JM (2001). Fish fauna associated with drifting seaweed in the coastal area of Tongyeong, Korea. Trans Am Fish Soc.

[CR4] Hernández CG, Hughes B, Graham MH (2006). Reproductive longevity of drifting kelp *Macrocystis pyrifera* (Phaeophyceae) in Monterey Bay, USA. J Phycol.

[CR5] Ikehara K (2004). Seasonal variations and distribution of floating seaweeds in Japan Sea. Kaiyo Monthly.

[CR6] Kawamata S (2001). Adaptive mechanical tolerance and dislodgement velocity of the kelp *Laminaria japonica* in wave-induced water motion. Mar Ecol Prog Ser.

[CR7] Komatsu T, Mikami A, Matsunaga D (2006). Distribution of floating seaweeds in East China Sea. Kaiyo Monthly.

[CR8] Komatsu T, Tatsukawa K, Filippi JB, Sagawa T, Matsunaga D, Mikami A, Ishida K, Ajisaka T, Tanaka K, Aoki M, Wang WD, Liu FL, Zhang SY, Zhou MD, Sugimoto T (2007). Distribution of drifting seaweeds in eastern East China Sea. J Mar Syst.

[CR9] Komatsu T, Mikami A, Ajisaka T, Uwai S, Aoki M, Tanaka K, Fukuda M, Kokubu Y, Tanaka K, Michida Y, Sugimoto T (2009). Ecological characteristics of drifting seaweed rafts composed of *Sargassum* species. Bull Coast Oceanogr.

[CR10] Komatsu T, Mizuno S, Alabsi N, Kantachumpoo A, Tanaka K, Morimoto A, Hsiao ST, Rothäusler EA, Shishidou H, Aoki M, Ajisaka T (2014). Unusual distribution of floating seaweeds in the East China Sea in the early spring of 2012. J Appl Phycol.

[CR11] Komatsu T, Ohtaki T, Sakamoto S, Sawayama S, Hamana Y, Shibata M, Shibata K, Sasa S (2015) Impact of the 2011 Tsunami on seagrass and seaweed beds in Otsuchi Bay, Sanriku Coast, Japan. In: Ceccaldi H-J, Hénocque Y, Koike Y, Komatsu T, SDtora G, Tusseau-Vuillemin M-H (eds), Marine Productivity: Perturbations and Resilience of socio-ecosystems. Springer, pp 43–53

[CR12] Koumoto Y, Tomiyama A (1968). Collection of spores and their culture using synthetic fibers as collector. Aquacult Sci.

[CR13] Kuwahara H, Kaneta T, Kawai T (1999). Attachment limits on the substratum for adult and sporophytes of *Cystoseira hakodatensis* (Yendo) Fensholt under roles of waves. Proc Int Sess, Coast Eng J.

[CR14] Marui M, Inai S, Yoshida T (1981). Growth and maturation of six species of *Sargassum* and *Cystoseira* (Phaeophyta, Fucales) in Oshoro Bay, Hokkaido, Japan. Jpn J Phycol.

[CR15] Mikami A (2007) Estimating the net primary production of *Sargassum* species (Phaeophyceae) throughout different life stages: from fixation to drifting. Dissertation, The University of Tokyo, Tokyo. (in Japanese with English abstract)

[CR16] Milligan KLD, DeWreede RE (2000). Variations in holdfast attachment mechanics with developmental stage, substratum-type, season and wave-exposure for the intertidal kelp species *Hedophyllum sessile* (C.Agardh) Setchell. J Exp Mar Biol Ecol.

[CR17] Mizuno S, Ajisaka T, Lahbib S, Kokubu Y, Alabsi MN, Komatsu T (2014). Spatial distributions of floating seaweeds in the East China Sea from late winter to early spring. J Appl Phycol.

[CR18] Okuda T (1985). Obtaining germlings of some *Sargassum* species and their settlement mechanisms. Kaiyo Monthly.

[CR19] Rueness J (1989). *Sargassum muticum* and other introduced Japanese macroalgae. Mar Pollut Bull.

[CR20] Senta T (1986). Mechanism that juvenile fishes accompany drifting seaweeds. Kaiyo Monthly.

[CR21] Stewart HL (2006). Morphological variation and phenotypic plasticity of buoyancy in the macroalga *Turbinaria ornata* across a barrier reef. Mar Biol.

[CR22] Sugawara A, Seto M, Komatsu T (1998). Study on the zonation of macro algae: vertical distribution and current environments in *Eisenia bicycli*s Setchell and *Ecklonia cava* Kjellman. Proc Civil Eng Ocean.

[CR23] Sun JZ, Chen WD, Zhuang DG, Zheng HY, Lin L, Pang SJ (2008). In situ ecological studies of the subtidal brown alga *Sargassum horneri* (Turner) C. Agardh at Nanji Island of China. S China Fish Sci.

[CR24] Tahara M (1913). Oogonium liberation and the embryogeny of some fucaceous algae. J Coll Sci, Imperial Univ Tokyo, Jpn.

[CR25] Tala F, Gómez I, Luna JG, Thiel M (2013). Morphological, physiological and reproductive conditions of rafting bull kelp (*Durvillaea antarctica*) in northern-central Chile (30°S). Mar Biol.

[CR26] Terawaki T (1986). Growth and maturation of *Sargassum horneri* (Turner) C. Agardh in Odawa Bay, Miura Peninsula. Suisanzoshoku.

[CR27] Thiel M, Gutow L (2005). The ecology of rafting in the marine environment. 1. The floating substrata. Oceanogr Mar Biol Ann Rev.

[CR28] Umezaki I (1984). Ecological studies of *Sargassum horneri* (Turner) C. Agardh in Obama Bay, Japan Sea. Bull Jpn Soc Sci Fish.

[CR29] Yoshida T (1963). Studies on the distribution and drift of the floating seaweed. Bull Tohoku Reg Fish Res Lab.

